# Novel Insights on the Bacterial and Archaeal Diversity of the Panarea Shallow-Water Hydrothermal Vent Field

**DOI:** 10.3390/microorganisms11102464

**Published:** 2023-09-30

**Authors:** Erika Arcadi, Emanuela Buschi, Eugenio Rastelli, Michael Tangherlini, Pasquale De Luca, Valentina Esposito, Rosario Calogero, Franco Andaloro, Teresa Romeo, Roberto Danovaro

**Affiliations:** 1 Department of Integrative Marine Ecology, Stazione Zoologica Anton Dohrn, Sicily Marine Centre, Contrada Porticatello, 29, 98167 Messina, Italy; erika.arcadi@szn.it (E.A.); rosario.calogero@szn.it (R.C.); franco.andaloro@szn.it (F.A.); 2 Department of Marine Biotechnology, Stazione Zoologica Anton Dohrn, Fano Marine Centre, Viale Adriatico 1-N, 61032 Fano, Italy; emanuela.buschi@szn.it; 3 Department of Research Infrastructures for Marine Biological Resources, Stazione Zoologica Anton Dohrn, Fano Marine Centre, Viale Adriatico 1-N, 61032 Fano, Italy; 4 Department of Research Infrastructures for Marine Biological Resources, Stazione Zoologica Anton Dohrn, Villa Comunale, 80121 Napoli, Italy; pasquale.deluca@szn.it; 5 Istituto Nazionale di Oceanografia e di Geofisica Sperimentale—OGS Borgo Grotta Gigante 42/C, 34010 Sgonico, Italy; vesposito@inogs.it; 6 Department of Biology and Evolution of Marine Organisms, Stazione Zoologica Anton Dohrn, Sicily Marine Centre, Via dei Mille 46, 98057 Milazzo, Italy; 7 National Institute for Environmental Protection and Research, Via dei Mille 46, 98057 Milazzo, Italy; 8 Department of Life and Environmental Sciences, Polytechnic University of Marche, Via Brecce Bianche, 60131 Ancona, Italy; r.danovaro@univpm.it; 9 National Biodiversity Future Centre (NBFC), 90133 Palermo, Italy

**Keywords:** next-generation sequencing, hydrothermal vent, benthic marine ecosystems, microbial assemblage diversity, turnover diversity

## Abstract

Current knowledge of the microbial diversity of shallow-water hydrothermal vents is still limited. Recent evidence suggests that these peculiar and heterogeneous systems might host highly diversified microbial assemblages with novel or poorly characterized lineages. In the present work, we used 16S rRNA gene metabarcoding to provide novel insights into the diversity of the bacterial and archaeal assemblages in seawater and sediments of three shallow-water hydrothermal systems of Panarea Island (Tyrrhenian Sea). The three areas were characterized by hot, cold, or intermediate temperatures and related venting activities. Microbial biodiversity in seawater largely differed from the benthic one, both in α-diversity (i.e., richness of amplicon sequence variants—ASVs) and in prokaryotic assemblage composition. Furthermore, at the class level, the pelagic prokaryotic assemblages were very similar among sites, whereas the benthic microbial assemblages differed markedly, reflecting the distinct features of the hydrothermal activities at the three sites we investigated. Our results show that ongoing high-temperature emissions can influence prokaryotic α-diversity at the seafloor, increasing turnover (β-)diversity, and that the intermediate-temperature-venting spot that experienced a violent gas explosion 20 years ago now displays the highest benthic prokaryotic diversity. Overall, our results suggest that hydrothermal vent dynamics around Panarea Island can contribute to an increase in the local heterogeneity of physical–chemical conditions, especially at the seafloor, in turn boosting the overall microbial (γ-)diversity of this peculiar hydrothermal system.

## 1. Introduction

Hydrothermal vents can be found in all oceans, even at shallow depths. These systems represent extreme environments where high temperature is associated with reduced and low pH emissions, enriched in heavy metals and toxic compounds, and select specific taxa adapted to these conditions [1]. The analysis of these systems can contribute to expanding our understanding of the adaptation strategies that organisms use to cope with extreme environmental conditions [2]. Hydrothermal vents are common in deep-sea areas (volcanic areas and mid-ocean ridges) but can be present also at shallow-water depths [3,4,5]. Indeed, although hydrothermal vents at shallower water depths are abundant worldwide, easier to access, and spatially closer to human life than their deep-sea analogs, knowledge of their microbial diversity and functioning is still scarce [6,7,8,9]. They are situated within the photic zone where light still reaches the seafloor and are characterized by high biological productivity due to complex microbial communities of photosynthetic, chemosynthetic, or mixotrophic microbes that use sunlight and/or hydrothermal energy for their metabolic needs [10,11,12,13,14]. Here, microorganisms are involved in the transformation of chemical compounds released from the vent emissions and are at the basis of the hydrothermal system food web; hence, their study is essential to understanding hydrothermal biogeochemical cycles [9,15,16].

From the limited number of available studies on microbial diversity of the shallow-water hydrothermal vents, it is often difficult to draw general conclusions, as different studies used different sampling and sequencing approaches. Yet, current information highlights that Gammaproteobacteria and Campylobacteria (formerly Epsilonproteobacteria) [17] are usually the most common bacterial taxa that drive carbon, sulfur, and nitrogen cycling in both vent fluids and sediments [2,4,6,9,18,19]. Several investigations have also highlighted that microbial communities associated with shallow-water vents can markedly change both spatially and/or temporarily [20,21]. If the hydrothermal venting is reduced, hence decreasing the available chemical energy, the microbial assemblages can rapidly change, for instance, by shifting to photosynthetic lifestyle and related taxa [9,15]. Indeed, vent prokaryotic microbes usually show ample metabolic flexibility, which fosters their adaptation to these particularly unstable and rapidly evolving ecosystems [18,22].

The hydrothermal field off Panarea Island is the most active vent area of the entire Mediterranean Sea [16,19]. Panarea vents have mainly been described to occur at shallow-water depths (<200 m depth), although the hydrothermal activity is known to have effects down to the deep sea (i.e., >400 m depth), including the presence of solid deposits, chimneys, and significant amounts of heavy metals [16]. The emitted gases have been described to consist mostly of CO_2_ and variable concentrations of H_2_S, CH_4_, CO, and H_2_ [16]. Previous studies in this area reported metabolically and taxonomically diverse bacterial and archaeal taxa with a patchy distribution in the surface sediments [13,19,23].

In this work, we conducted a high spatial scale resolution study to provide novel insights into the diversity of the microbial assemblages in the seawater and surface sediments of this shallow-water hydrothermal system. We investigated three sites characterized by a gradient of venting emissions: two sites (Hot Vent and Cold Vent) within an area characterized by either hot or cold vent fluid emissions, as well as one site showing intermediate venting conditions (i.e., Bottaro Crater Vent). In November 2002, this latter area experienced a violent fluid emission, which added further environmental heterogeneity within the Panarea hydrothermal vent field [16,24,25]. In this study, we thus also investigated the potential effect of this explosive event on the resident microbial assemblages.

## 2. Materials and Methods

### 2.1. Sampling Sites and Sampling Strategy

**Figure 1:**
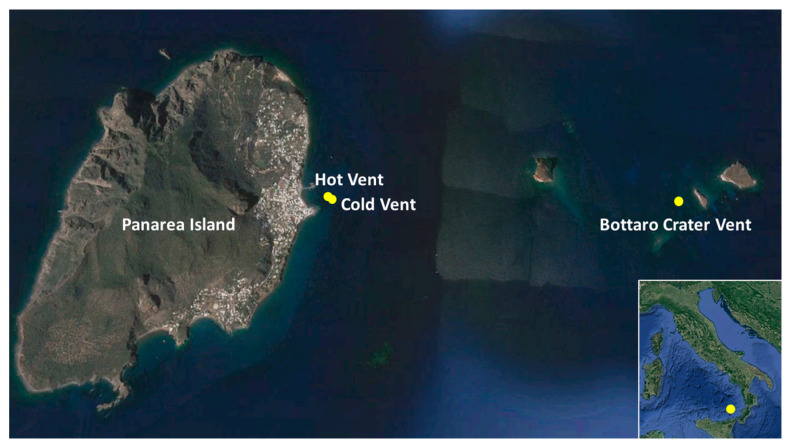
The figure shows the map of the study area, with the location of the Panarea vent sites (Hot, Cold, and Bottaro Crater Vents) sampled in the present study. The map was generated using Google Earth Pro, version 7.3.6, available at the following URL: https://www.google.it/earth/about/versions/, accessed on 15 May 2023.

The samples’ collection was carried out in September 2019 in three shallow-water sites of the Panarea hydrothermal vent field: the Hot Vent (38°38′022.95″ N; 15°04′045.62″ E), the Cold Vent (38°38′022.97″ N; 15°04′045.67″ E), and the Bottaro Crater Vent (38°38′013.58″ N; 15°6′033.95″ E), (Table S1; Figure 1). At each site, we collected bottom seawater samples with previously sterilized 4L Niskin bottles, as well as surface sediment samples (top 2 cm) with sterilized Plexiglas core tubes, manually operated by SCUBA diving. Overall, we collected three seawater samples (one at each vent site) and four sediment samples (one at the Hot Vent, one at the Cold Vent, and two at the Bottaro Crater Vent). The main chemical–physical parameters were measured at all sites through a multi-parametric probe (CTD profiler, SBE 19 Plus SeaCAT probe), and pH was determined spectrophotometrically, as has been reported by [8] (and replicated in Table S1 for ease of visualization).

### 2.2. DNA Extraction and Purification and Downstream Molecular Analyses

Two liters of bottom water from each site were filtered on a 47 mm nitrocellulose filter 0.22 µm pore size and stored at −20 °C until DNA extraction. Total DNA was extracted and purified from filters by using the GNOME DNA kit (MP Biomedicals, Medical Systems SPA, Genoa, Italy) and from 0.5 g of each sediment sample by using FastDNA SPIN Kit for Soil (MP Biomedicals, Medical Systems SPA, Genoa, Italy) according to the kit’s standard protocols. The obtained DNA samples were eluted in 50 μL of TE buffer, and their quality and concentration were determined by 0.6% agarose gel electrophoresis, using Lambda DNA/HindIII Marker (ThermoFisher Scientific, Rome, Italy) and by spectrophotometric measurements using a NanoDrop^®^ ND-1000 Spectrophotometer (ThermoFisher Scientific, Rome, Italy). Ribosomal 16S amplifications were performed on a BioRad C1000 touch thermocycler (BioRad Laboratories, Naples, Italy) in a final volume of 25 µL. Primers were modified at their 5′-ends adding two short tails of 18 (forward primer) and 17 (reverse primer) nucleotides in length subsequently used as anchorage for the second PCR to ligate barcodes and P1 sequences, respectively [26]. The following mix was used: 1 µL extracted DNA was added to 10 µL PCR buffer, 0.5 µM of each of the two primers (515F–Y 5′-TAIL18-GTGYCAGCMGCCGCGGTAA; and 926R 5′-TAIL17-CCGYCAATTYMTTTRAGTTT; [27]), and 1.25U/reaction PrimeStar GXL DNA polymerase (Takara Bio Inc, Diatech Lab Line, Jesi, Italy). The PCR cycles were characterized by an initial denaturation of 5 min at 98 °C, followed by 35 cycles of 10 sec at 98 °C, 15 sec annealing at 53 °C, and 35 sec extension at 68 °C, with final extension of 7 min at 68 °C. All amplification products were run on a 1% agarose-TAE gel to verify the size of the amplicons and then purified with the AMPure XP magnetic beads (Beckman Coulter, Venice, Italy), following manufacturer’s instructions. The second PCR amplification (switch PCR) [26] was performed using the same volumes of reagents as in the first PCR but replacing the primer cocktail with Barcodes and P1 solution [26] at a final concentration of 0.5 μM each and using approximately 5 ng of the first PCR product as template and 1.25U/reaction PrimeStar GXL DNA polymerase (Takara Bio Inc, Diatech Lab Line, Jesi, Italy) in a final volume of 25 μL. The thermal profiling for the switch PCR started at 98 °C for 2 min, followed by 5 cycles of denaturation at 98 °C for 10 s, annealing at 60 °C for 15 s, and extension at 68 °C for 40 s, with a final extension at 68 °C for 7 min. PCR products were purified with the AMPure XP magnetic beads (Beckman Coulter, Venice, Italy), following manufacturer’s instructions. The quantity and quality of the PCR products were determined with the Agilent DNA High Sensitivity Kit on the Bioanalyzer (Agilent Technologies Italia Spa, Milan, Italy) following the manufacturer’s recommendations. An equimolar amount of all the libraries were pooled, following Thermo Fisher indications. The quantity of DNA was assessed using the dsDNA HS Assay Kit on the Qubit 3.0 fluorimeter (Thermo Fisher Scientific, Rome, Italy) following the manufacturer’s protocol. Library amplification and enrichment and chip loading were performed in automation using the Ion S5 ExT Chef Reagents and the Ion chip 530 on the Ion Chef system (Thermo Fisher Scientific, Rome, Italy) following the manufacturer’s recommendations. Massive parallel sequencing was carried out on the Ion GeneStudio S5 System with the Ion S5 ExT Sequencing Reagents (Thermo Fisher Scientific, Rome, Italy) at the SZN Sequencing and Molecular Analysis Center. Raw data were analyzed by Torrent Suite (Thermo Fisher, Scientific, Rome, Italy) to obtain BAM files to be used in downstream bioinformatic analyses.

### 2.3. Bioinformatic and Statistical Analyses

The raw single-end sequences were imported into the QIIME2 pipeline, and their quality was checked using the tools provided by the suite [28]; leftover primer sequences were identified and removed through the *cutadapt* plugin before running the DADA2 denoising procedure on the high-quality sequences using default parameters, after sequence trimming at 300 bp, following cutoff recommendations for single-end ION Torrent sequencing and including removal of singletons [29]. A subset of the SILVA database (v.138.1) [30] was produced by trimming the original database on the region amplified by the primer set through the QIIME2 extract-reads procedure; this subset was used as a reference for taxonomic affiliation of representative amplicon sequence variants (ASVs), which was carried out through the classify-consensus-vsearch classifier, with standard parameters. After ASV classification, ASVs matching eukaryotic, mitochondrial, and chloroplast sequences, as well as those without any classification, were removed to produce a reliable taxonomic table [29]. To allow the comparison of all samples despite differences in sequencing depths, we rarefied the resulting ASV table at the lowest number of sequences across the dataset (n = 160,000 sequences) by running the core-metrics procedure, which was also utilized to calculate alpha-diversity values and produce beta-diversity distance matrices [29]. ASV profile comparisons were performed using the statistical packages within the STAMP program (using standard two-sided White’s non-parametric *t*-test, DP: bootstrap 0.95, and Bonferroni correction) [31], and the number of ASVs exclusive of single samples or shared between and among samples was obtained through the VennDiagram package starting from the results of the ASV table rarefaction analysis. SIMPER analyses were performed to assess the turnover diversity as percentage of dissimilarity in prokaryotic assemblage composition [32,33,34]. To test the effects of environmental variables on prokaryotic assemblages, non-parametric multivariate multiple regression analyses based on Bray–Curtis distances were carried out using distance-based linear model (DISTLM) routine with the *forward* selection procedure. All statistical tests were performed using the PRIMER v6 + PERMANOVA add-on software [35,36].

## 3. Results and Discussion

**Figure 2:**
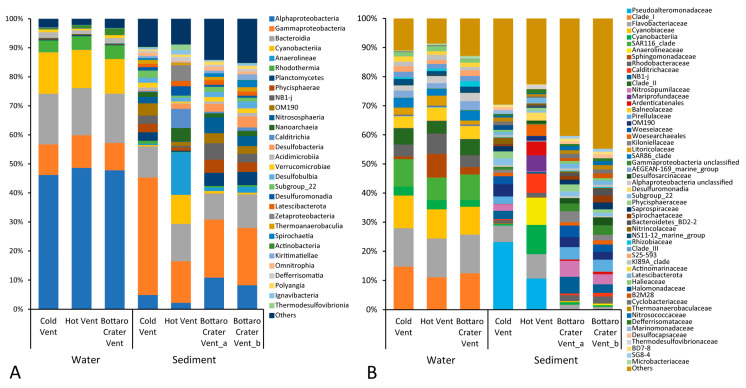
Bar plots showing the taxonomic composition of prokaryotic assemblages in each bottom seawater sample and sediment sample collected at the different sites investigated in the present study at class level (**A**) and family level (**B**). The bar colors reflect the taxonomic color legend next to each plot.

The 16S rRNA gene metabarcoding analysis we carried out exhaustively covered the prokaryotic assemblage diversity within the samples collected in this study (Figure S1). The taxonomic annotation of the prokaryotic ASVs allowed us to identify a total of 168 taxa if aggregating at the class level and 546 taxa if aggregating at the family level. Sediment samples displayed the most diversified assemblages, with 167 class-level taxa and 505 family-level taxa, while the bottom water samples were composed of only 95 class-level taxa and 351 family-level taxa. In water samples, the most abundant bacterial class was Alphaproteobacteria, followed by Bacteroidia, Cyanobacteria, and Gammaproteobacteria classes (on average, 48%, 17%, 13%, and 10%, respectively), with similar contributions in the three water samples we investigated (Figure 2).

The Classes Alphaproteobacteria, Cyanobacteria, Bacteroidia, and Rhodothermia together accounted for approximately 50% of the total diversity in water samples, mainly represented by Clade I, Clade II, Clade III, SAR116, Cyanobiaceae, Flavobacteraceae, and Balneolaceae. These were otherwise almost absent in the sediments (<0.2% Figure S2). Conversely, the bacterial classes OM190, Subgroup22, and Phycisphaerae, as well as the archaeal classes Nitrosospheria and Nanoarcheia, were relatively abundant in sediment samples (each comprising from 2% to 12% of the whole assemblage), otherwise remaining below 0.3% in the seawater samples (Figure S2).

These results confirm the high taxonomic dissimilarity between the prokaryotic assemblages of bottom waters, as well as their remarkable difference with respect to sediment samples. Such difference between planktonic and benthic microbial assemblages has been frequently reported in other vent and non-vent marine ecosystems [15,37]. On the other hand, the prokaryotic assemblage composition found in the water samples analyzed here largely differs from previous investigations in the fluids/bottom waters of the Panarea hydrothermal vent [13,23] or of other pelagic sites at shallow-water hydrothermal vents [1,18]. In fact, the families reported in this study from water samples (see also the taxonomic analysis at the genus level in Table S3) mostly include common autotrophic and heterotrophic bacteria typical of non-extreme pelagic marine ecosystems [38,39,40,41]. This finding might suggest that the hydrothermal activity at the time of our sampling was too limited to influence the bottom-water microbial assemblages of the investigated sites.

**Figure 3:**
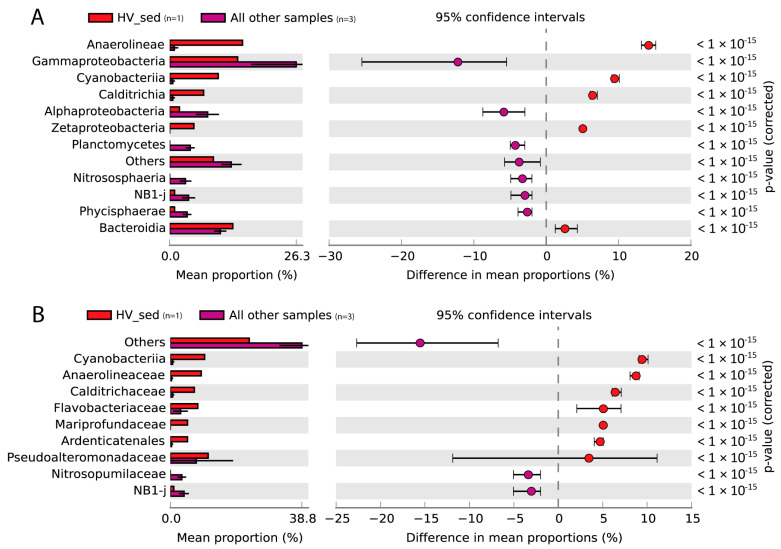
Bar plots and extended error bar plots showing the main prokaryotic taxa responsible for the significant differences between Hot Vent sediment (HV_sed) and the other sediment samples investigated in the present study at class (**A**) and family (**B**) levels. Sample size is indicated in parenthesis next to the color legend.

**Figure 4:**
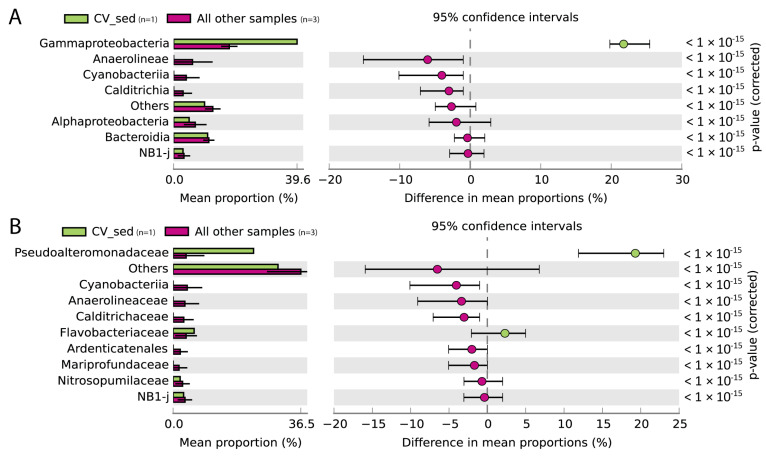
Bar plots and extended error bar plots showing the main prokaryotic taxa responsible for the significant differences between Cold Vent sediment (CV_sed) and the other sediment samples investigated in the present study at class level (**A**) and at family level (**B**). Sample size is indicated in parenthesis next to the color legend.

**Figure 5:**
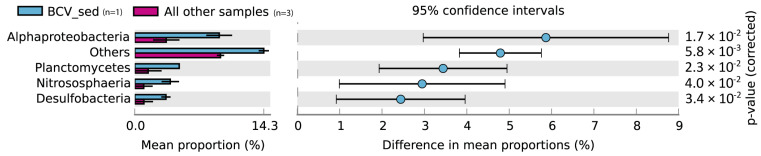
Bar plot and extended error bar plot showing the main prokaryotic taxa responsible for the significant differences between Bottaro Crater Vent sediment (BCV_sed) and the other vent sediment samples investigated in the present study. Sample size is indicated in parenthesis next to the color legend.

The most abundant bacterial class in the sediment samples was Gammaproteobacteria, though with variable relevance in the different vents (14% in Hot Vent, 20% in Bottaro Crater, and 40% in Cold Vent), followed by Bacteroidia (13% in Hot Vent, 10% in Bottaro Crater, and 11% in Cold Vent) and Alphaproteobacteria (2% in Hot Vent, 9% in Bottaro Crater, and 5% in Cold Vent) (Figure 2). Whilst water samples were taxonomically similar at the three investigated sites at both class and family levels, the sediment samples showed evident differences (Figure 3, Figure 4 and Figure 5).

Such results were confirmed by the DISTLM analysis, which highlighted temperature and pH as significant drivers of the prokaryotic assemblage diversity in the analyzed samples (overall accounting for >79% of the total variation; *p* < 0.05; Table S2; Figure S4).

The sediments of Hot Vent were characterized by much larger relative abundances of Anaerolineae, Cyanobacteria, Calditrichia, Zetaproteobateria, Desulfuromonadia, and Nanoarchaeia (mainly represented by Anaerolineaceae, Calditrichaceae, and Mariprofundaceae, as well as unclassified Ardenticatenales, Cyanobacteria, Desulfuromonadia, and Woesearchaeales), that were otherwise almost absent in the Cold Vent and Bottaro Crater Vent sediments (Figure 2 and Figure 3).

Anaerolineaceae and Calditrichaceae have been reported as key bacterial taxa of shallow-water hydrothermal vents, involved not only in organic carbon remineralization but also in nitrogen cycling [15,42,43], and include moderate thermophilic chemoheterotrophs, which grow by fermentation of peptides or carbohydrates compounds and have been described as anaerobic digesters in several organic-rich habitats [44,45,46]. Analogously, bacteria within the orders Ardenticantales and Woesearchaeales (ammonium-oxidizing archaea) are known as key components of the microbial assemblages at hydrothermal vents, also participating in C, N, and especially Fe cycling [47,48,49]. In this regard, the reported presence of particularly high Fe concentration in the Panarea sediments [50] can explain the local proliferation of bacteria whose metabolic functions are strictly dependent upon Fe(III)/Fe(II) redox reactions. This was confirmed by the identification of abundant mat-forming, autotrophic Fe-oxidizing Mariprofundacea bacteria (Figure 2 and Table S3). These bacteria, together with the Fe-reducing Desulfuromonadia, may contribute to the wide orange-red mats that characterized the Hot Vent sediments and suggest intense iron biogeochemistry and mineral deposition in the investigated hydrothermal system [51,52,53].

Such taxonomic features largely differ from those previously reported in other Panarea or Vulcano Island hot vents, in which sulphur-oxidizing bacteria belonging to Campylobacterota (previously, Epsilonproteobacteria), such as Sulfurimonas, Arcobacter and Sulfurovum spp. contributed to >50% of the total microbial diversity [19,54]. Indeed, despite the primers we used for 16S rRNA gene sequencing widely targeting this taxon (>95% coverage as checked using SILVA TestPrime; www.arb-silva.de/search/testprime/; accessed on 20 September 2023 [30]), Campylobacterota was not detected from the Hot Vent site of our study (and only slightly represented also in the other samples we investigated; Figure 2). Conversely, our study indicates that the cyanobacteria were largely (>10 times) overrepresented at the Panarea sites characterized by hot vent fluid emissions. This finding is consistent with previous reports from this area reporting an abundant presence of the thermophilic and mixotrophic Synecoccoccus genus [13].

The Cold Vent sediments, even though at only a few-meter distance from the Hot Vent site, displayed a very different benthic prokaryotic assemblage, dominated by Gammaproteobacteria largely represented by *Pseudoalteromonas* spp. (accounting for 23% of the Cold Vent sediment total reads alone, i.e., 2–20 times more than in Hot Vent and Bottaro Crater Vent sediments, respectively) (Figure 2 and Figure 4; Table S3).

Pseudoalteromonadaceae include aerobic, heterotrophic, and extremophilic bacteria with wide metabolic and physiologic versatility. This taxon includes producers of biofilms and exopolysaccharides, which underlie their well-known adaptability to a wide range of peculiar environments such as extremely cold habitats, deep-sea sediments, and hydrothermal vents [15,55,56]. The Pseudoalteromonas spp. flourishing in the Cold Vent site may be favored by the more acidic pH conditions that characterize the sediments around the Panarea Hot/Cold Vents (~1 pH unit lower than at the Bottaro Crater), being, at the same time, able to tolerate the episodic intrusion of hot fluids from the hot vents nearby.

The Flavobacteraceae bacterial family showed higher relative abundances in the Cold Vent and Hot Vent sediments than in the Bottaro Crater sediments (6% and 8% vs. <1%, respectively), and its taxonomic composition largely differed between the Cold and Hot Vent. Indeed, whilst Cold Vent sediments hosted *Sediminicola*, *Zeaxanthinibacter*, *Nonlabens*, and *Aquibacter* spp., which include strictly aerobic, heterotrophic marine bacteria largely involved in organic matter degradation [57,58], Hot Vent sediments only comprised unclassified Flavobacteraceae (Table S3), suggesting that high-temperature marine ecosystems like the Hot Vent spot we investigated may be a source of novel members of this bacterial family, whose metabolic features remain to be studied.

We also report that Alphaproteobacteria, Planctomycetes, Desulfobacteria, and Nitrososphaeria were largely over-represented in the sediments from the Bottaro Crater Vent than in those of the Hot Vent and Cold Vent sites (Figure 5).

These bacterial and archaeal taxa are usually more abundant in sediments not interested in hydrothermal activity and typically decrease when approaching the hot vent fluids [14,19,43,54]. This agrees well with the results reported here for the Bottaro Crater spot investigated, which experienced a major gas explosion in November 2002 and now displays a much weaker, milder temperature of venting, with temperatures much lower than other hot vent sites of the Panarea hydrothermal vent field [16]. Previous studies conducted recently near the Bottaro Crater (at the Black Point site, also affected by the same gas explosion of November 2002) reported similarly high abundances of non-vent microbial taxa [13,23], corroborating our conclusion that this site shows intermediate features between vent and non-vent ecosystems in terms of prokaryotic assemblages.

These results suggest that the sudden change in environmental conditions caused by the past gas explosion at the Bottaro Crater Vent site probably led to a striking diversification of the benthic environmental niches available for prokaryotes. This likely contributed to generating physical–chemical ecotones and ecoclines across both time and space in this area [59,60], allowing the co-existence of microbes typical of both vent and non-vent benthic ecosystems in a relatively small area and turning the Bottaro Crater Vent site into a peculiar hot-spot of microbial biodiversity.

**Figure 6:**
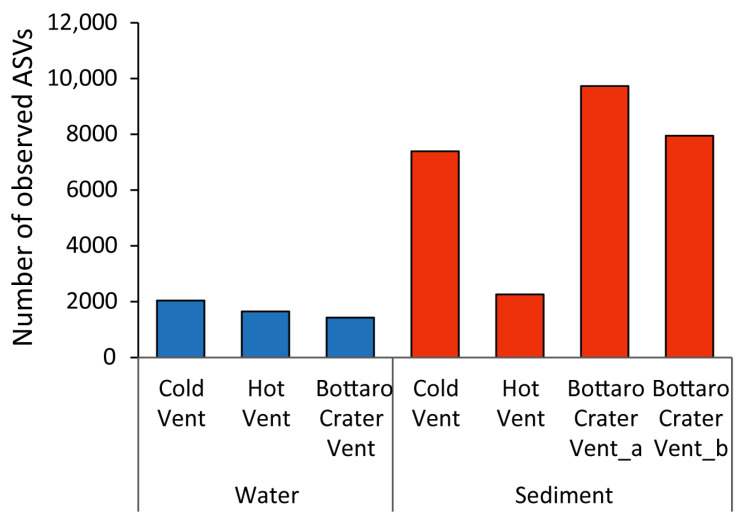
Bar plot showing values of ASV richness of prokaryotic assemblages in each bottom seawater sample and sediment sample collected at the different hydrothermal vent sites investigated in the present study.

This conclusion was also confirmed by the analysis conducted at the ASV level, which revealed high variability in ASV richness (α-diversity) (Figure 6). The Bottaro Crater Vent sediments (on average, 8863 ASVs) showed the highest values, followed by the Cold Vent sediments (7423 ASVs) and Hot Vent sediments (2230 ASVs). Water samples reported ASV richness values up to four times lower compared to sediments (on average, 1549 ASVs), whose trend was expected, as frequently reported by comparative studies of marine water vs. sediment samples in both vent and non-vent environmental settings [13,15]. The particularly high ASV richness values detected at the Bottaro Crater Vent sediments are consistent with previous findings for this specific site [13,19,23]. Conversely, the lower prokaryotic diversity found in the sediment of the Hot Vent (Table S1) confirms that high-temperature vent fluids can locally decrease the prokaryotic ASV richness, as previously observed in other vent sites also [19,43,61].

As anticipated, microbial diversity at the ASV level was distinct (~100% dissimilarity) between planktonic and benthic prokaryotic assemblages (Figure S3). We found a large dissimilarity (≥90% turnover β-diversity) among benthic prokaryotic assemblages, while planktonic prokaryotic assemblages were relatively more similar to each other (41–46% turnover β-diversity) (Figure S3), indicating that the planktonic prokaryotic assemblages were more homogeneous in terms of ASV composition than the benthic ones.

**Figure 7:**
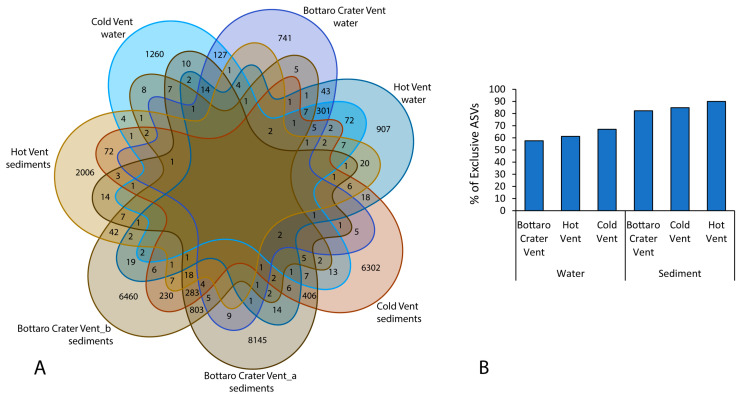
Venn diagram showing the shared and exclusive ASVs among all the samples collected from the sites investigated in the present study (**A**), and bar plot showing the distribution of exclusive ASVs across the different samples investigated in the present study (**B**).

Out of a total of 28501 ASVs (all samples), none were shared by all samples, 301 ASVs were shared only among seawater samples, and only 18 were shared among all sediment samples (Figure 7A).

Overall, the number of exclusive ASVs (i.e., ASVs found in a single sample category only) increased from plankton to benthic assemblages (58–67% of exclusive ASVs) and further tendentially increased in benthic samples while moving towards the Hot Vent site (82% at the Bottaro Crater Vent, 85% at the Cold Vent, and 90% at the Hot Vent sites; Figure 7A,B).

**Figure 8:**
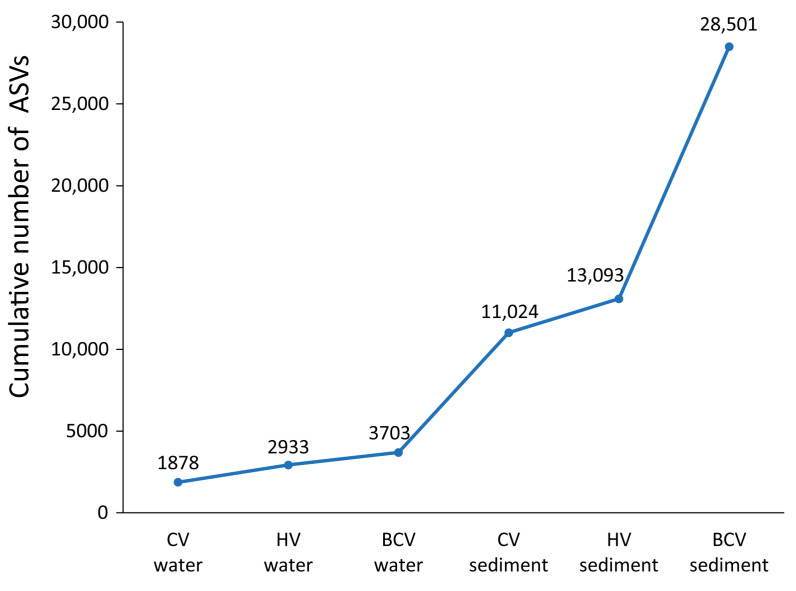
Linear plot showing the cumulative contribution of each sample category to the total (γ-)diversity (at ASV level) of the Panarea shallow-water hydrothermal sites investigated in the present study. The solid line is drawn to emphasize the difference in the contribution between the different sample categories.

The analysis of the cumulative prokaryotic ASV richness (γ-diversity) highlighted that the Bottaro Crater sediments contributed the major portion (54%) to the total biodiversity (expressed as ASV richness; Figure 8).

This suggests that microbial (as Bacteria and Archaea) biodiversity, particularly in benthic assemblages, is promoted by the high heterogeneity either in terms of geological–mineralogical–topographic features and on venting gradients, reaching the highest values in areas where we observe the co-existence of vent and non-vent conditions.

## 4. Conclusions

Here, we provided new insights into the diversity of the microbial assemblages inhabiting seawater and sediments from three shallow-water hydrothermal vent sites near Panarea Island (Tyrrhenian Sea). We provide evidence of a distinct taxonomic composition between the planktonic and benthic microbial assemblages and that the hot vent fluids can select the bacterial and archaeal taxa, resulting in a decrease in benthic microbial diversity. Each vent site showed a distinct taxonomic composition of the benthic prokaryotic assemblages, indicating that the high heterogeneity of hydrothermal features at the Panarea vent promotes microbial turnover (β-)diversity. The Bottaro Crater Vent site, created by past gas explosions and presently characterized by milder venting features compared to the Hot Vent, displayed mixed vent/non-vent features in terms of prokaryotic taxonomic profiles and the highest diversification at the ASV level. As also reported by previous independent studies on other marine hydrothermal sites [13,19,20,21,23], our data suggest that the high heterogeneity of the seafloor (also in terms of physical–chemical conditions) related to hydrothermal activity around Panarea Island supports a high microbial (γ-)diversity. We conclude by highlighting the need to conduct further investigations by increasing the replication level and using comparable sequencing pipelines across studies to reach more definitive and robust conclusions on the patterns and drivers of prokaryotic diversity in this and other shallow-water vent sites.

## Data Availability

The datasets presented in this study were deposited in online repositories. The name of the repository and accession numbers can be found in the Figshare repository (URL: 10.6084/m9.figshare.22841903, accessed on 15 May 2023).

## Supplementary Materials

The following supporting information can be downloaded at https://www.mdpi.com/article/10.3390/microorganisms11102464/s1, Table S1. Environmental characterization of the different sampling sites of Cold Vent, Hot Vent and Bottaro Crater Vent within the Panarea Shallow Hydrothermal system; Table S2. Outputs of the statistical analysis of the DISTLM marginal tests carried out on the dataset of the taxonomic composition of the prokaryotic assemblages at (a) ASV, (b) family and (c) class level in the seawater and sediment samples of the different vent sites; Figure S1. Rarefaction curves of the observed ASVs in the 3 different vent sites at increasing sequencing depth; Figure S2. Taxonomic composition of the prokaryotic assemblages in water and sediment samples. Panel A shows a heatmap to highlight the clustering of the samples based on the relative abundance of the main prokaryotic classes identified; in panel B, the main prokaryotic classes responsible for the significant differences between water and sediment samples are shown; in panel C, higher resolution (at family level) analysis of the main prokaryotic taxa responsible for the significant differences between water and sediment samples; Figure S3. PCoA plot showing the turnover (β-)diversity in water and sediment samples of the three different vent sites. % values indicate the percent dissimilarity at ASV level among samples; Figure S4. dbRDA showing the relationship between environmental variables and the taxonomic composition of prokaryotic assemblages (at family level) of the bottom waters and sediments of the three vent sites investigated. Table S3: excel file showing results of the taxonomic analysis at the genus level.
