# Supplementary material for: Novel Insights on the Bacterial and Archaeal Diversity of the Panarea Shallow-Water Hydrothermal Vent Field
Source: Microorganisms. 2023 Sep 30;11(10):2464. doi: 10.3390/microorganisms11102464 (PMC10608945; doi:10.3390/microorganisms11102464)
Supplement: Supplementary file 1 [file microorganisms-11-02464-s001.zip › Arcadi et al._Supplementary materials.pdf]

Supplementary materials for

Novel insights on the bacterial and archaeal diversity of the Panarea shallow-water hydrothermal vent field

Erika Arcadi, Emanuela Buschi, Eugenio Rastelli, Michael Tangherlini, Pasquale De Luca, Valentina Esposito, Rosario Calogero, Franco Andaloro, Teresa Romeo, and Roberto Danovaro

This file includes:

Supplementary Tables: **Table S1**, **Table S2**

Supplementary Figures: **Figure S1-S4**.

Table S1. Environmental characterization of the different sampling sites of Cold Vent, Hot Vent and Bottaro Crater Vent within the Panarea Shallow Hydrothermal system.

Location	Source samples	Depth (m)	Temperature (°C)	pH
Cold Vent	Bottom seawater	11	26.2	7.89
	Sediment	11	26	5.91
Hot Vent	Bottom seawater	11.4	25.39	8.07
	Sediment	11.7	48	5.54
Bottaro Crater Vent	Bottom seawater	7.5	19.26	8.17
	Sediment	7.5	28	6.78

Table S2. Outputs of the statistical analysis of the DISTLM marginal tests carried out on the dataset of the taxonomic composition of the prokaryotic assemblages at (a) ASV, (b) family and (c) class level in the seawater and sediment samples of the different vent sites.

**(a) Prokaryotes assemblages at ASV level**				
Variable	SS(trace)	Pseudo-F	P	Prop.
Temperature	5895,7	1,5313	0,010	0,23446
pH	8903,3	2,7407	0,021	0,35406

**(b) Prokaryotes assemblages at family level**				
Variable	SS(trace)	Pseudo-F	P	Prop.
Temperature	2606,4	1,911	0,046	0,27652
pH	6241,2	9,7983	0,025	0,66212

**(c) Prokaryotes assemblages at class level**				
Variable	SS(trace)	Pseudo-F	P	Prop.
Temperature	1213,3	1,9337	0,049	0,27888
pH	2780	8,8496	0,032	0,63898

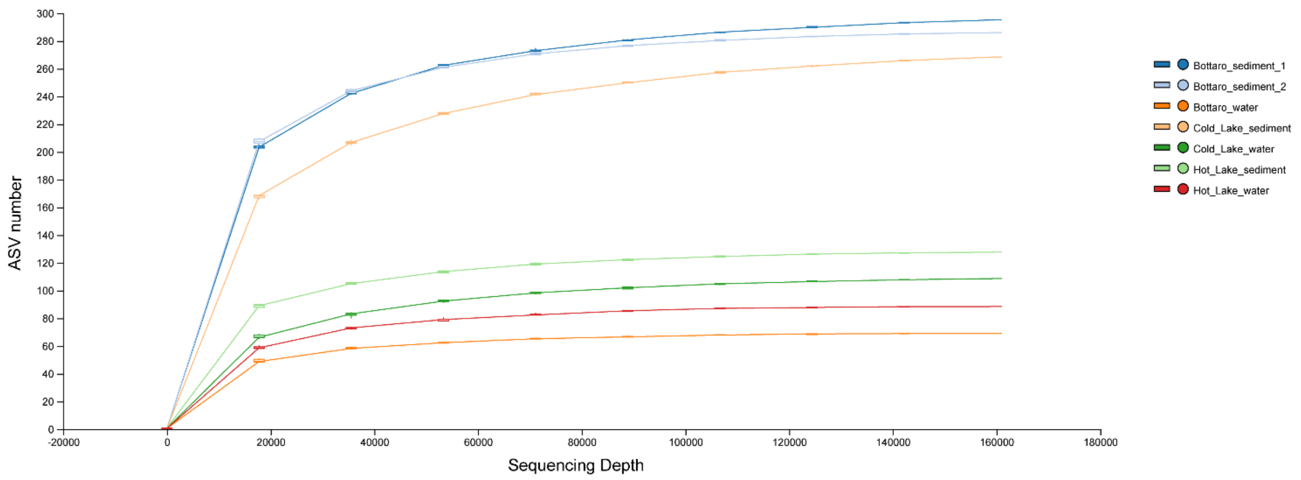

Figure S1. Rarefaction curves of the observed ASVs in the 3 different vent sites at increasing sequencing depth.

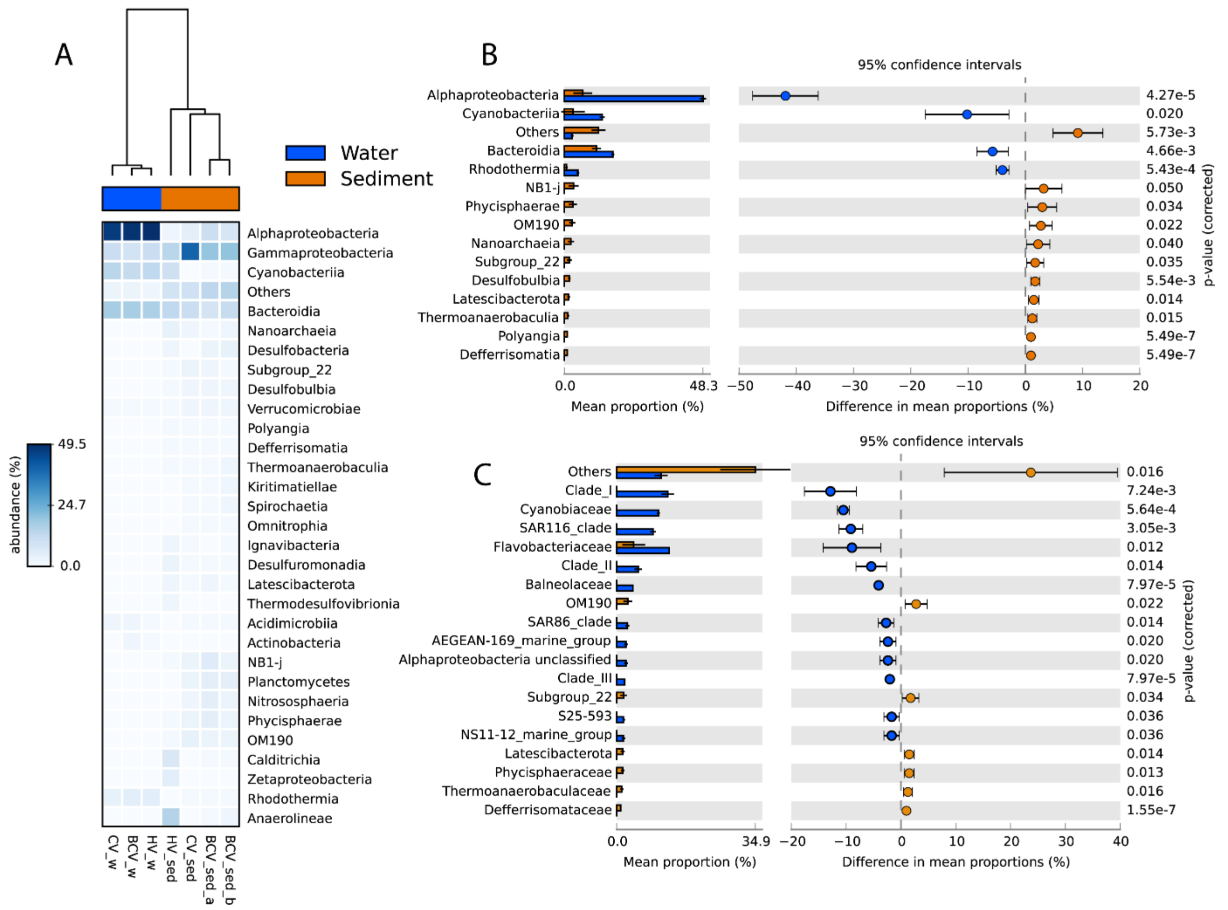

Figure S2. Taxonomic composition of the prokaryotic assemblages in water and sediment samples. Panel A shows a heatmap to highlight the clustering of the samples based on the relative abundance of the main prokaryotic classes identified; in panel B, the main prokaryotic classes responsible for the significant differences between water and sediment samples are shown; in panel C, higher resolution (at family level) analysis of the main prokaryotic taxa responsible for the significant differences between water and sediment samples.

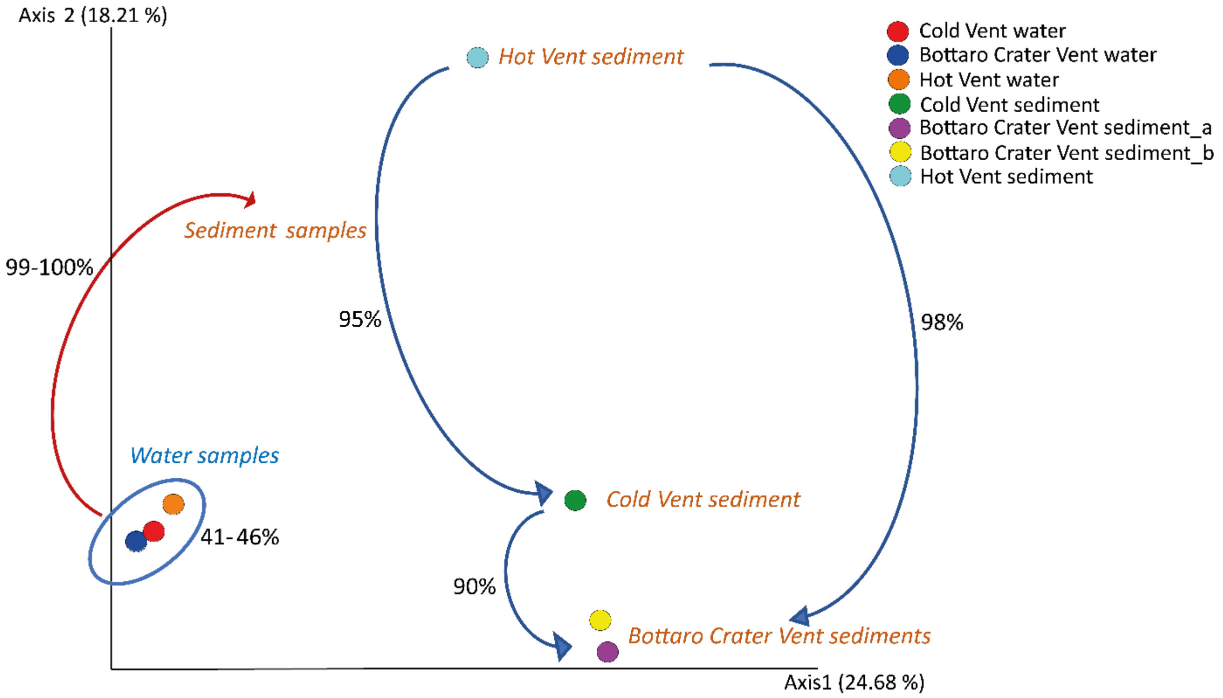

Figure S3. PCoA plot showing the turnover (β-)diversity in water and sediment samples of the three different vent sites. % values indicate the percent dissimilarity at ASV level among samples.

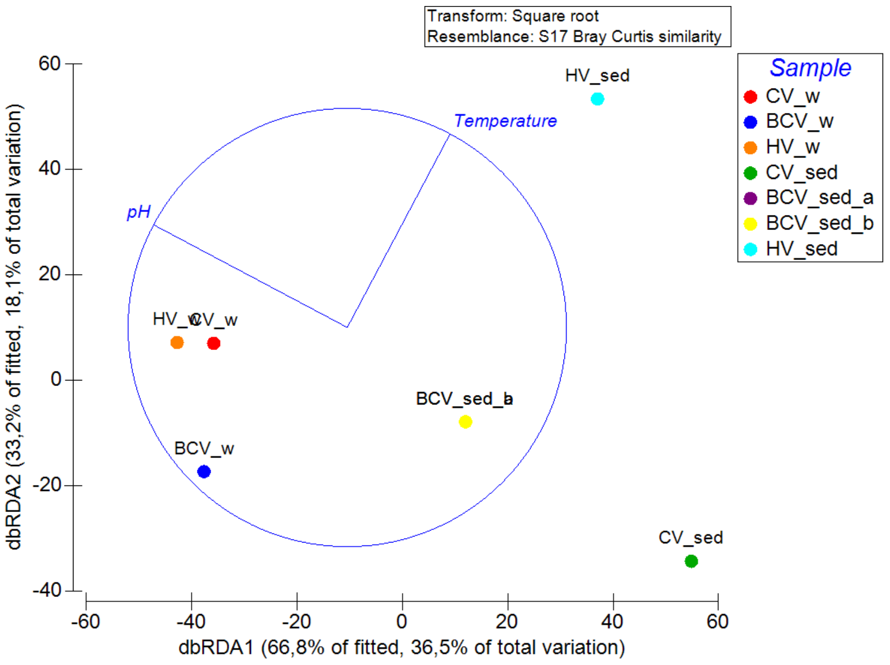

Figure S4. dbRDA showing the relationship between environmental variables and the taxonomic composition of prokaryotic assemblages (at family level) of the bottom waters and sediments of the three vent sites investigated.
